# Novel phylogenetic clade of avian *Haemoproteus* parasites (Haemosporida, Haemoproteidae) from Accipitridae raptors, with description of a new *Haemoproteus* species[Fn FN1]

**DOI:** 10.1051/parasite/2023066

**Published:** 2024-02-08

**Authors:** Josef Harl, Anaïs Fauchois, Marie-Pierre Puech, Delphine Gey, Frédéric Ariey, Brigitte Izac, Herbert Weissenböck, Nayden Chakarov, Tatjana Iezhova, Gediminas Valkiūnas, Linda Duval

**Affiliations:** 1 Institute of Pathology, Department of Pathobiology, University of Veterinary Medicine Vienna Veterinaerplatz 1 1210 Vienna Austria; 2 Département Adaptations du Vivant (AVIV), Molécules de Communication et Adaptation des Microorganismes (MCAM, UMR 7245 CNRS), Muséum National d’Histoire Naturelle, CNRS, CP 52 57 rue Cuvier 75231 Cedex 05 Paris France; 3 Hôpital de la faune sauvage des Garrigues et Cévennes – Goupil Connexion 34190 Brissac France; 4 Université de Paris, INSERM 1016, Institut Cochin, Service de Parasitologie-Mycologie Hôpital Cochin Paris France; 5 Department of Animal Behaviour, Bielefeld University Konsequenz 45 33615 Bielefeld Germany; 6 Nature Research Centre Akademijos 2 08412 Vilnius Lithuania

**Keywords:** Accipitridae, *Haemoproteus*, New species, *cytb* DNA barcoding, Birds, New phylogenetic clade

## Abstract

Avian haemosporidian parasites (order Haemosporida, phylum Apicomplexa) are blood and tissue parasites transmitted by blood-sucking dipteran insects. Three genera (*Plasmodium*, *Haemoproteus* and *Leucocytozoon*) have been most often found in birds, with over 270 species described and named in avian hosts based mainly on the morphological characters of blood stages. A broad diversity of *Haemoproteus* parasites remains to be identified and characterized morphologically and molecularly, especially those infecting birds of prey, an underrepresented bird group in haemosporidian parasite studies. The aim of this study was to investigate and identify *Haemoproteus* parasites from a large sample comprising accipitriform raptors of 16 species combining morphological and new molecular protocols targeting the *cytb* genes of this parasite group. This study provides morphological descriptions and molecular characterizations of two *Haemoproteus* species, *H. multivacuolatus* n. sp. and *H. nisi* Peirce and Marquiss, 1983. *Haemoproteus* parasites of this group were so far found in accipitriform raptors only and might be classified into a separate subgenus or even genus. *Cytb* sequences of these parasites diverge by more than 15% from those of all others known avian haemosporidian genera and form a unique phylogenetic clade. This study underlines the importance of developing new diagnostic tools to detect molecularly highly divergent parasites that might be undetectable by commonly used conventional tools.

## Abstract (fr)

Les parasites hémosporidies aviaires (ordre Haemosporida, phylum Apicomplexa) sont des parasites sanguins et tissulaires transmis par des insectes diptères hématophages. Trois genres (*Plasmodium*, *Haemoproteus* et *Leucocytozoon*) ont été le plus souvent trouvés chez les oiseaux, avec plus de 270 espèces décrites et nommées chez les hôtes aviaires en fonction principalement des caractères morphologiques des stades sanguins. Une grande diversité des *Haemoproteus* reste à identifier et à caractériser morphologiquement et génétiquement, en particulier ceux qui infectent les oiseaux de proie, un groupe d’oiseaux sous-représenté dans les études sur les hémosporidies. Le but de cette étude était d’étudier et d’identifier les *Haemoproteus* à partir d’un large échantillon comprenant des rapaces accipitriformes de 16 espèces, en combinant des protocoles morphologiques et de nouveaux protocoles moléculaires ciblant les gènes *cytb* de ce groupe de parasites. Cette étude fournit des descriptions morphologiques et des caractérisations moléculaires de deux espèces d’*Haemoproteus*, *H. multivacuolatus* n. sp. et *H. nisi* Peirce and Marquiss, 1983. Les *Haemoproteus* de ce groupe n’ont jusqu’à présent été trouvés que chez les rapaces accipitriformes et pourraient être classés dans un sous-genre ou même un genre distinct. Les séquences *cytb* de ces parasites divergent de plus de 15 % de celles de tous les autres genres d’hémosporidies aviaires connus et forment un clade phylogénétique unique. Cette étude souligne l’importance de développer de nouveaux outils de diagnostic pour détecter des parasites moléculairement très divergents qui pourraient être indétectables par les outils conventionnels couramment utilisés.

## Introduction

Avian haemosporidian parasites (order Haemosporida, phylum Apicomplexa) are blood and tissue parasites transmitted by blood-sucking dipteran insects [53]. They are diverse, widespread and have been reported in birds of most orders [13, 48]. The three genera *Plasmodium*, *Haemoproteus*, and *Leucocytozoon* include the majority of haemosporidian parasites found in birds, with over 270 species described based mainly on the morphological characters of blood stages [14, 22].

The introduction of DNA barcoding protocols, mainly targeting a 478 bp fragment of the mitochondrial *Cytochrome B* (*cytb*) gene [6, 20], has revealed genetic diversity much higher than previously assumed based on the number of described morphospecies. Over 4000 unique *cytb* lineages have been listed in the MalAvi database (Avian Malaria Initiative, http://130.235.244.92/Malavi/) [7]. DNA barcoding of the *cytb* revealed a high diversity of cryptic species, which are either morphologically indistinguishable or have not been described yet [41, 53]. Moreover, it showed that submicroscopic infections (not detected by microscopy) and co-infections (several parasites of different species simultaneously infecting the same bird individual) are common in birds [25, 42].

The genus *Haemoproteus* is the most diverse genus of avian haemosporidian parasites with more than 170 described species and many new *Haemoproteus* morphospecies being continuously described and characterised molecularly [1, 55, 57]. In addition, almost half of the *cytb* lineages available in the MalAvi database belong to the genus *Haemoproteus*, but only about 160 lineages have been linked to approximately 75 *Haemoproteus* morphospecies [33, 54].

The genus *Haemoproteus* includes two subgenera, *Haemoproteus* and *Parahaemoproteus*, whose species can be distinguished by their life history traits [4, 5, 30, 53]. The most distinctive characteristics of species of the subgenus *Haemoproteus* are the pattern of sporogony (development of numerous germinal centres in oocysts) and the transmission by louse flies (Hippoboscidae). The species of the subgenus *Parahaemoproteus* are more widely distributed, have a different pattern of sporogony (only one germinal centre occurs in oocysts), and are transmitted by biting midges (Ceratopogonidae).

The studies addressing the life cycles and vectors of *Haemoproteus* parasites remain few [2, 23, 62]. Identification and description of *Haemoproteus* parasites are usually done using morphological characteristics of their blood stages (gametocytes) and patterns of their influence on host cells. This approach remains the reference method for species identification, together with molecular characterization using the 478 bp *cytb* barcode sequence for haemosporidian parasites [8, 58].

Today, the taxonomic status of the genus *Haemoproteus* remains valid but has been challenged by some studies, *e.g.*, the discovery of parasites of the subgenus *Haemoproteus* in several species of seabirds has changed the understanding of the parasite-host associations for this subgenus, which historically has been assumed exclusive to Columbiformes [3, 31, 35]. Furthermore, a recent publication investigating *Haemoproteus* parasites infecting cranes [9], exemplary of the parasites of understudied avian orders, revealed a novel *Haemoproteus* clade, which might be classified as a separate genus or subgenus in future studies, but currently, the biology of these parasites remains completely under-investigated.

The knowledge regarding the morphological and molecular diversity of avian *Haemoproteus* parasites is still limited and biased by heterogeneous sampling efforts in terms of bird species and geographic regions investigated. Most sampling has focused on passerine birds (75% of currently available DNA sequence data), while haemosporidian parasites of other bird orders [14] are far less investigated.

A broad diversity of *Haemoproteus* parasites remains undescribed and numerous *Haemoproteus* morphospecies need to be identified and characterized molecularly [58]. The phylogenetic position of these parasites within the phylogeny of Haemosporida remains unresolved [11, 15, 52]. Further studies focusing on parasites of underrepresented bird groups are required to solve taxonomic and systematic issues. The development of new PCR protocols is necessary to estimate the diversity of *Haemoproteus* parasites because the widely used PCR protocol developed by Hellgren *et al.* [19] does not amplify the *cytb* barcode sequence of some *Haemoproteus* and *Leucocytozoon* parasites [18, 54, 60].

Accipitriform birds are an underrepresented bird group compared to the passerines in haemosporidian parasite studies [38, 42, 45, 50]. However, they represent a good model group for the study of blood parasites because they are widespread across large geographical areas, found in a variety of habitats, and commonly delivered in wildlife rescue centres. They are ecologically sensitive apex predators and valuable indicators of habitat quality [28, 37, 39].

Recent studies on haemosporidian parasites of accipitriform raptors in Europe (Austria, Bosnia-Herzegovina, and Czechia) revealed a large diversity of new *cytb* lineages belonging to the three genera *Plasmodium*, *Haemoproteus*, and *Leucocytozoon*, suggesting that the diversity of haemosporidian parasites is greater than previously assumed, and many parasite lineages remain non-described or have not yet been linked to known morphospecies [18, 51]. Furthermore, phylogenetic analyses revealed that *cytb* lineages of *Haemoproteus* parasites specific to accipitriform birds form a phylogenetically distant group [51], which might represent a separate group at the subgenus or genus levels within the family Haemoproteidae [18].

Three *Haemoproteus* species were found in Accipitridae raptors from various geographic regions in Europe, using the traditional taxonomy. These are *H. elani* Mello, 1935, *H. nisi* Peirce and Marquiss, 1983 and *H. buteonis* Wingstrand, 1947 [44, 53, 61]. None of these three *Haemoproteus* morphospecies has been characterized molecularly to date.

The aim of this study was to investigate and identify *Haemoproteus* parasites from a large sample comprising accipitriform raptors of 16 species combining morphological and molecular approaches. Most samples originated from *Buteo buteo* (Common buzzard), *Accipiter nisus* (Eurasian sparrowhawk), and *Circus aeruginosus* (Western marsh harrier), three of the most common accipitriform raptors in Eurasia and Africa [37]. A new multiplexed PCR amplicon-based next-generation sequencing approach was developed specially to investigate the molecular diversity of *Haemoproteus* lineages parasitizing accipitriform birds. In addition, the complete mitochondrial genomes of *H. multivacuolatus* n. sp. hBUBT1 and *H. nisi* hACCNIS06 were sequenced.

## Material and methods

### Ethics statement

The samples from France collected by Dr. Marie-Pierre Puech, Doctor of Veterinary Medicine, wild animal health center (Hôpital de la Faune Sauvage) Goupil connexion, were taken for routine examinations. Only surplus blood samples were used in this study.

The collection of blood samples from living birds in Austria was approved by the institutional ethics and animal welfare committee and the national authority according to §§ 26ff. of the Animal Experiments Acts, Tierversuchsgesetz TVG 2012, Austria (BMWFW- 68.205/0036-WF/V/3b/2017). The samples collected by the Research Institute of Wildlife Ecology (University of Veterinary Medicine Vienna) and the Faculty of Veterinary Medicine (University of Sarajevo) were taken from carcasses submitted for routine examinations. No birds were injured or killed for the present study.

The collection of blood samples from Germany was approved by the Animal Ethics Committee at Bielefeld University and conducted with permission from the local authority Kreis Gütersloh, permit number: 4.5.2–723-Bussard and from the federal state authority 84–02.04.2014.A091, 84–02-04.2017.A147 in accordance with German federal and state laws.

### Biological samples

The samples from France comprised 413 diurnal raptors of the family Accipitridae: 277 *Buteo buteo*, 131 *Accipiter nisus*, and 5 *Circus aeruginosus*. In all, 77/413 were found to be infected with *Haemoproteus* parasites after microscopic examinations and chosen for molecular identification. All birds were admitted for post-injury care to the wild animal health center (Hôpital de la Faune Sauvage, Languedoc-Roussillon, Ganges), sampled upon admission for routine veterinary purposes from 2012 to 2021 and released after recovery. Blood sample surpluses were used to prepare two thin blood smears which were air-dried and the remaining blood was put in EDTA tubes. All thin blood smears were fixed with absolute methanol prior to Giemsa staining (8% in phosphate buffered solution) for 45 min, covered with cover slips, and mounted with Eukitt^®^ resin for long term storage. All blood samples in EDTA tubes were frozen until molecular analysis.

The samples from Austria comprised 171 birds of the order Accipitriformes: 9 *Accipiter gentilis*, 22 *A. nisus*, 1 *Aquila chrysaetos*, 10 *Aquila heliaca*, 70 *Buteo buteo*, 1 *Buteo lagopus*, 5 *Buteo* sp., 22 *Circus aeruginosus*, 4 *Circus cyaneus*, 2 *Clanga pomarina*, 2 *Gypaetus barbatus*, 1 *Gyps fulvus*, 12 *Haliaeetus albicilla*, 8 *Milvus milvus*, 1 *Pandion haliaetus*, and 1 *Pernis apivorus*. The Research Institute of Wildlife Ecology (Department of Interdisciplinary Life Sciences, Vetmeduni Vienna) provided frozen tissue samples of 113 birds collected between 2009 and 2018 in Austria. Blood samples were taken from 58 living birds received for treatment at the service unit for birds and reptiles of the clinic for small animal internal medicine (Department for Companion Animals and Horses, Vetmeduni Vienna) between 2015 and 2016. The blood was taken by puncturing the brachial vein using heparinized microcapillaries to transfer blood drops to high-grade filter papers Whatman™ 903 (GE Healthcare, Amersham, UK), and two blood smears were prepared as described above for a subset of individuals kept at the clinic. The samples were already screened molecularly for haemosporidian parasites of other groups by Harl *et al.* [18].

The samples from Germany were collected from 832 *Buteo buteo* nestlings in North Rhine-Westphalia and Lower Saxony, between 2019 and 2022. The samples were collected from late May to early July from nestlings ranging between 2 and 6 weeks of age as described in Chakarov *et al.* [12]. Blood was taken with a syringe from the ulnar vein to prepare smears, which were fixed and Giemsa stained, and the rest of the blood was stored in absolute ethanol. The samples included in this study were found to be BUBT1-positive through PCR screening as described below and microscopic screening.

### Microscopic examination and parasite morphometry

The blood smears were carefully examined for parasite identification with a motorised BX63 Upright Olympus Microscope at a magnification of 1000 under oil immersion. *Haemoproteus* parasites were photographed with an Olympus DP72 Camera (High-Speed 12.8 Megapixel Image Capture) and morphologically identified according to Valkiūnas [53, 58]. Morphometric measurements were performed on 21 fully grown gametocytes using the cellSens Dimension 1.9 software and the imaging software analySIS FIVE (Olympus Soft Imaging Solution GmbH, Münster, Germany). Parasitaemia was calculated by actual counting of the number of gametocytes per 1 000 erythrocytes. Student’s t-test for independent samples was used to determine statistically significant differences between mean linear parameters. A *p*-value of 0.05 or less was considered significant.

### DNA extraction

Total genomic DNA of the samples from France was extracted from 20 μL of whole blood samples using a QIAamp DNA Micro Kit (QIAGEN, Courtaboeuf, France), following the manufacturer’s instructions for isolation of genomic DNA from small volumes of blood. DNA of the samples from Austria was extracted either from tissue (liver and spleen) or blood spots using a DNeasy Blood & Tissue Kit (QIAGEN, Venlo, Netherlands), following the manufacturer’s protocol for isolation of DNA from tissue samples. DNA from samples from Germany was extracted from blood *via* a common Phenol-Isopropanol-Ethanol extraction protocol.

### PCR and sequencing of the *cytb* barcode region

All samples from France were screened for haemosporidian parasites using a nested PCR protocol targeting a 478 bp fragment of the mitochondrial *Cytochrome B* (*cytb*) of *Haemoproteus* spp. and *Plasmodium* spp., which is commonly used as a DNA barcode sequence [6, 19]. These samples were screened with a nested PCR assay using the newly designed primers H1 (5′–TGG TAC TAC AGG AGT AAT GTT AGG–3′) and H2 (5′–CGT CTA AGC ATG TTA ACT CGA TTG–3′) to amplify a 1670 bp amplicon, followed by two nested-PCRs using two sets of primers also newly designed FSP1F (5′–GAA TTAT GGA RTG GAT GGT G–3′) and FSP1R (5′–GCT GTA TCA TAY CCT AAA GG–3′) and ACC2F (5′–GGA TTT GTG GTG GAT ATA G–3′) and ACC2R (5′–GGA GTC ACA AAT ARA CTA AC–3′) to amplify two shorter amplicons of 415 bp and 303 bp, respectively. These amplicons together encompassed the entire *cytb* barcode region (478 bp length) with 153 bp overlap.

The samples from Austria were previously screened with the nested PCR protocol by Hellgren *et al.* [19] and a second nested PCR protocol specifically targeting parasites of the *Leucocytozoon toddi* group [18]. For the present study, these samples were screened with a nested PCR assay specifically targeting the *cytb* of BUBT1 and related parasite lineages. The newly designed primers CytB_Hnis_F1 (5′–GGA GTA CTA CTT GCT ACC AGA T–3′) and CytB_Hnis_R1 (5′–GTT TGC TTG GG AGC TGT AAT C–3′) were used to amplify a 1000 bp amplicon, and CytB_Hnis_F2 (5′–TCA CCA GAA ATG GAT TAT GC–3′) and CytB_Hnis_R2 (5′–TGT GGT AAT GTA GAT CCT ATC C–3′) were used in a nested PCR to amplify an 863 bp section (821 bp excluding primers) of the *cytb* including the entire DNA barcode region.

The samples from Germany were screened with a one-round PCR reaction and newly designed primers specifically targeting BUBT1 and related parasite lineages. These primers BUBT1_F3 (FAM-labelled 5′–CGG GCA GAT GAC AGA AAC TAT–3′) and BUBT1_R3 (5′–TTT GCC TGG AGG TTA TGT TCT–3′) were used to amplify a 680 bp fragment in the non-coding mitochondrial region of BUBT1, while being multiplex-compatible with other primers designed to score other blood parasite lineages found in common buzzards (Chakarov *et al.*, in prep). A selection of samples was lineage-verified by amplification and Sanger-sequencing with the same primers.

The PCRs of the samples from France were performed in a final volume of 25 μL consisting of 5 μL of 5× PrimeSTAR GXL Buffer, 2 μL of dNTP Mixture (2.5 mM each), 0.5 μL of each primer (0.2 μM), 0.5 μL PrimeSTAR GXL DNA Polymerase (1.25U) (Takara Bio, Shiga, Japan), 15 μL of nuclease-free water and 2 μL of DNA template. The PCRs included denaturation at 98 °C for 10 s, followed by 40 cycles of amplification at 98 °C for 10 s, 60 °C for 15 s, 68 °C for 2 min (PCR reaction) or 45 s (nested-PCR reaction), and a final extension at 68 °C for 10 min. The PCR products were visualised on 2% agarose gel. The nested PCRs of the samples from Austria were performed with a KAPA2G Fast HotStart PCR kit (Sigma Aldrich, St. Louis, MO, USA) in 25 μL volumes containing 12.5 μL polymerase mix, 8.5 μL nuclease-free water, 1 μL MgCl (25 mM), each 1 μL primer, and 2 μL template. The PCR cycles included denaturation at 94 °C for 2 min, followed by 25 (first PCR)/35 (nested PCR) cycles at 94 °C for 30 s, 54 °C (first PCR)/50 °C (nested PCR) for 30 s, 72 °C for 1 min, and a final extension at 72 °C for 10 min. One μL of the first PCRs was used as template in the nested PCRs. The PCR products were visualised on 1% agarose gels.

For sequencing the PCR products of the samples from France, a dual-indexing approach was developed to allow high-throughput sequencing of multiplexed 478 bp *cytb* barcode amplicons on Illumina MiSeq [49]. Thus, 13 primer tags of 5 nucleotides were designed and added to the 5′ end of nested PCR primers to form 40 unique combinations. Thus, two sets of primer tags were used: AGTCT, ATTGC, ACGTC, ATGCG, AGATC, ATCTG, CATTG, CTAGG for forward nested PCR primers, and CGGAT, CGTGA, CTGTA, CGATT, TTGAC for reverse nested PCR primers, respectively. Tagged primers were synthetised with HPLC purification by Eurofins Genomics. The generated amplicons were indexed with unique 5-mer tags from both directions. Tagged amplicons were pooled into two series of 38 and 39 samples, respectively in a 1.5 mL Eppendorf tube and purified using 1× Ampure XP beads (Beckman Coulter). A second indexing step was carried out during the preparation of the two libraries with the NEBNext UltraII DNA Library Prep Kit for Illumina (New England BioLabs, Ipswich, MA, USA). The preparation includes an end-prep (blunt-end repair, 5′ phosphorylation and dA-tailing) from 1 μg of purified amplicons, a ligation step to attach Illumina adapters, a cleanup (0.9× Ampure XP beads) and PCR enrichment with indexing primers from NEBNext Multiplex Oligos for Illumina Set (3 cycles). A last purification step was performed on both libraries before checking their size distribution on an Agilent Bioanalyzer High Sensitivity DNA chip (Agilent Technologies, Vienna, Austria) and quantifying with a Qubit dsDNA HS assay kit (Invitrogen, ThermoFisher Scientific, Waltham, MA, USA). Both libraries were pooled at an equimolar ratio and sequenced using a 500 cycles Nano Kit v2 (2 × 250 bp, paired-end sequencing) on an Illumina MiSeq at iGenSeq platform, at ICM (CHU Pitié-Salpêtrière, Paris, France). The tagged amplicons were demultiplexed based on their unique tag combination using a customised python script. FastqR1 and fasqtR2 paired reads from both amplicon 1 and 2, respectively, were combined into single sequences using fastq-join Linux command and then aligned against a panel of 478 bp *cytb* sequences retrieved from MalAvi database [7] and the three complete *cytb* gene sequences from *Haemoproteus* of Accipitridae obtained in this study using Burrows-Wheeler Aligner (BWA) software [32]. The alignments were visualised using the Integrative Genomics Viewer IGV v2.13.2 [46].

The PCR products of the samples from Austria were sent to Microsynth Austria (Vienna, Austria) for purification and sequencing in both directions using the nested PCR primers. The raw forward and reverse sequences were aligned and visually inspected with BioEdit v. 7.0.5.3 [16].

The single round PCR-scoring of the samples from Germany was performed with a Type-it microsatellite PCR kit (QIAGEN) in 10 μL reaction volumes containing 5 μL Taq master mix, 3 μL water, 1 μL primer mix with 50 μM concentration for every included primer, and 1 μL template. The cycling conditions included an initial cycle of 95 °C for 5 min, and 35 cycles at 95 °C for 30 s, 57 °C for 30 s, and 72 °C for 60 s, followed by a final elongation round at 68 °C for 30 min. The resulting reaction volume was 1:20 diluted with water. The resulting fragments were resolved on an ABI 3730 Automated DNA Analyser using a 500 GeneScan LIZ ladder and analysed using GeneMarker 1.95 (SoftGenetics LCC, State College, PA, USA).

### PCR and sequencing of the complete *cytb* sequence

DNA samples containing single infections with three distinct *Haemoproteus* lineages were selected to amplify the whole *cytb* gene. The first PCR reactions were performed with the primers H1 (5′–TGG TAC TAC AGG AGT AAT GTT AGG–3′) and H2 (5′–CGT CTA AGC ATG TTA ACT CGA TTG–3′) to amplify a 1670 bp sequence. Nested PCRs were performed using the primers H3 (5′–ATG TAA TGC CTA GAC GTA TTC CTG–3′) and H4 (5′–CAT CCA TCA ACA GCT ATG GTA AC–3′) to amplify a 1369 bp amplicon including the complete *cytb* gene of each *Haemoproteus* lineage (hACCNIS06, hCIAE08 and hBUBT1). Libraries were prepared according to the protocol described by Meyer and Kircher [36] and sequenced using a 500 cycles Kit v2 (2 × 250 bp, paired-end sequencing) on an Illumina MiSeq Illumina at the Service de Systématique Moléculaire (SSM), part of the Service Unit Acquisition et Analyse de Données pour l’Histoire Naturelle (2AD) (UMS2700) at the Muséum National d’Histoire Naturelle in Paris.

FastqR1 and fastqR2 paired reads obtained from each sample were aligned against complete *cytb* reference sequences retrieved from GenBank (NCBI) and the MalAvi database [7] using Burrows-Wheeler Aligner (BWA) software [32]. The alignments were visualised using Integrative Genomics Viewer IGV v2.13.2 [46]. Reliable consensus *cytb* sequences from the three *Haemoproteus* parasite lineages were then manually generated.

### Whole mitochondrial genome captures and sequencing

Whole mitochondrial genome sequencing was performed only on hACCNIS06 and hBUBT1 lineages from two birds with single infections using HiSeq 2000 Illumina paired-read sequencing technology at the GENOM’IC Platform of Cochin Institute. Prior to sequencing, KAPA DNA library preparation and NimbleGen seqcap EZ haemosporidian DNA sequence capture, utilising specifically designed oligonucleotide 55- to 105-base DNA probes to isolate parasitic DNA, were performed using the SeqCap EZ HyperCap workflow, following the user guide (Roche).

Briefly, 500 ng of genomic DNA were sheared with a Covaris instrument, end-repaired, A-tailed and sequencing adapters were ligated to the fragments. The sample library was sized using Agencourt AMPure XP beads (±200–250 bp; Beckman Coulter Genomics) and enriched using 10 cycles of PCR before library quantification and validation using an Agilent 2100 Bioanalyser. Capture of haemosporidian DNA sequences was performed by hybridisation of the amplified sample library and the SeqCap EZ developer probes. The amplified enriched DNA library pool was then quantified using Agilent 2100 Bioanalyser and sequenced on Illumina HiSeq 2000 v2 kit (500 cycles, 2× 250 reads).

iSeGWalker Perl software was used to carry out a semi *de novo* genome reconstruction from fastqR1 and R2 paired reads data [27]. Short reads were then aligned against the mitochondrial genome sequence reconstructed as a reference using Burrows-Wheeler Aligner (BWA) software and also aligned against other mitochondrial genome references retrieved from the MalAvi database.

### DNA haplotype network

A DNA haplotype network was calculated with the 478 bp *cytb* barcode sequences of the new *Haemoproteus* lineages to visualise their relatedness and the distribution in different bird hosts. The network was solely based on the *cytb* sequences obtained from the Austrian and French raptors in the present study because the sequences published by other authors [24, 43] did not cover the entire DNA barcode region. A Median Joining haplotype network was calculated with Network 10.2.0.0 (Fluxus Technology Ltd, Suffolk, UK), using the default settings. The networks were graphically prepared and provided with information on host species and countries in Network Publisher v.2.1.2.5 (Fluxus Technology Ltd). The networks were then edited in Adobe Illustrator CC v.2015 (Adobe Inc., San Jose, CA, USA).

### Phylogenetic analysis

To assess the phylogenetic position of parasites belonging to the *Haemoproteus* species group of Accipitridae, a phylogenetic tree was calculated based on complete *cytochrome c oxidase 1* (*cox1*), *cytochrome c oxidase 3* (*cox3*), and *cytochrome b* (*cytb*) sequences of haemosporidian parasites. The original alignment contained all available complete mitochondrial genomes of haemosporidian parasites available on GenBank and the genomes of *H. multivacuolatus* hBUBT1 and *H. nisi* hACCNIS06, totalling 108 sequences. To reduce the data set, the sequences of ten unnamed mammalian *Plasmodium* species were excluded, resulting in an alignment with 98 haemosporidian genome sequences. The alignment was cut into three partitions containing the complete *cox1*, *cox3*, and *cytb* genes. *Klossiella equi* (MH203050) and *Klossia razorbacki* (MT084562) were included as outgroups because their mitochondrial genomes are most similar to those of haemosporidian parasites compared to other apicomplexan parasites. The sequences of the three partitions were aligned with MAFFT v.7 [26] using the default settings. The final alignments contained 1420 bp (*cox1*), 753 bp (*cox3*), and 1127 bp (c*ytb*). The substitution models were evaluated for all individual data sets using IQ-TREE v.1.6.12 [40]. According to the corrected Akaike information criterion (cAIC), the best substitution model for all alignments was GTR + I + G. Trees with both a partitioned data set and an unpartitioned data set (combining the three partitions in one alignment) were calculated, but the latter was selected because it resulted in higher support values for some nodes. A Maximum Likelihood (ML) “majority rule consensus” tree was calculated with IQ-TREE v.1.6.12 [40] by performing 10 000 bootstrap replicates. A Bayesian Inference (BI) tree was calculated with MrBayes v.3.2 [47]; the analysis was run for 5 million generations (2 runs with 4 chains, one of which was heated) and every thousandth tree was sampled. The first 25% of trees were discarded as burn-in and a 50% majority rule consensus tree was calculated from the remaining 3750 trees each.

In addition, ML and BI trees were calculated with *Babesia gibsoni* (AB499087) as outgroup. After removal of gap positions, the final alignments contained 1412 bp (*cox1*), 639 bp (*cox3*), and 1083 bp (*cytb*). The trees were calculated with the same settings as those calculated with *K. equi* and *K. razorbacki* as outgroups.

## Results

### Morphological characteristics of *Haemoproteus* parasites

Among French accipitriform raptor samples, 77/413 (18.6%) were positive for *Haemoproteus* parasites following microscopic examination of blood films, 53 *Buteo buteo*, 22 *Accipiter nisus*, and 2 *Circus aeruginosus*. These samples were therefore chosen for molecular screenings. In the case of the samples from Austria, the blood films were only available from a subset of individuals kept at the clinic. The samples from Germany originated from 832 nestlings sampled between 2019 and 2022. Blood smears and DNA were available for all samples. Among the German nestling samples, 69 were PCR-positive for hBUBT1 and therefore targeted for close microscopic examination.

The examination of all blood films revealed the presence of two distinct *Haemoproteus* morphospecies based on morphological characteristics of the gametocytes. The microscopy-positive samples that were confirmed positive for *Haemoproteus* spp. by PCR were then used for the molecular characterisation of the found parasite species.

The *Haemoproteus* parasites seen in *A. nisus* (lineage hACCNIS06) belonged to *H. nisi*. *Haemoproteus* parasites observed in blood films of *C. aeruginosus* (lineage hCIAE08) were morphologically similar to *H. nisi* but several minor morphological differences were visible; this parasite was considered a variant of *H. nisi*. *Haemoproteus* parasites observed in blood films of all PCR-positive and microscopically examined *B. buteo* from Germany, Austria, and France were morphologically similar and described as *Haemoproteus multivacuolatus* n. sp. Descriptions of the parasites found are given below.

**Figure 1:**
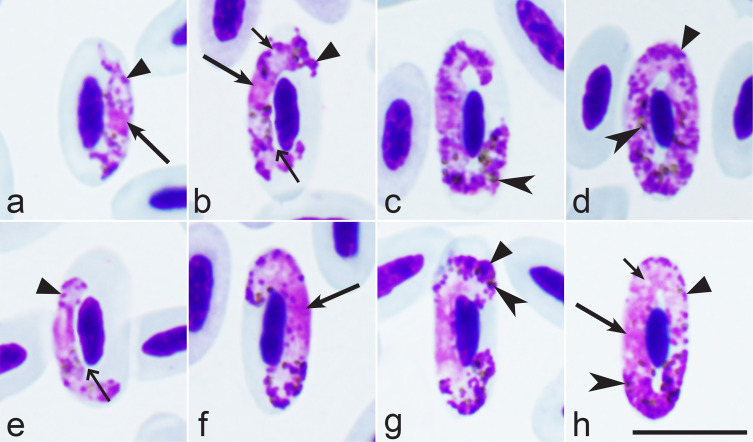
*Haemoproteus nisi* (lineage hACCNIS08) from the blood of Eurasian sparrowhawk *Accipiter nisus*: a–d – macrogametocytes, e–h – microgametocytes. Long simple arrows – nuclei of parasites. Short simple arrows – vacuoles. Simple arrowhead – pigment granules. Triangle arrowheads – volutin granules. Simple wide long arrows – spaces between gametocytes and erythrocyte nuclei. Giemsa-stained thin blood films. Scale bar = 10 μm.

Morphological characterisation of *H. nisi* Peirce and Marquiss, 1983, the parasite lineage hACCNIS06 found in the type host *A. nisus* (Figs. 1a–1h, Table 1)

**Table 1:** Morphometry of host cells and mature gametocytes of *Haemoproteus nisi* from *Accipiter nisus* (lineage hACCNIS06), *Circus aeruginosus* (hCIAE08), and *H. multivacuolatus* n. sp. (hBUBT1) from *Buteo buteo.*

Feature	Measurements (μm)^1^		
	*H. nisi* group		*H. multivacuolatus* n. sp.
	Lineage hACCNIS06	Lineage hCIAE08	Lineage hBUBT1
Uninfected erythrocyte			
Length	13.0–16.0 (14.6 ± 0.7)	12.6–15.8 (14.3 ± 05)	9.8–13.5 (11.4 ± 0.9)
Width	6.3–7.9 (6.7 ± 0.4)	6.0–7.7 (6.6 ± 0.3)	6.2–7.8 (6.8 ± 0.4)
Area	61.1–76.0 (69.6 ± 4.2)	59.8–79.0 (67.0 ± 5.0)	50.0–70.4 (62.0 ± 6.0)
Uninfected erythrocyte nucleus			
Length	6.4–8.4 (7.5 ± 0.5)	6.0–7.7 (6.5 ± 0.4)	4.5–5.7 (5.1 ± 0.3)
Width	2.1–2.7 (2.4 ± 0.2)	2.1–3.4 (3.1 ± 0.3)	3.0–4.3 (3.7 ± 0.3)
Area	10.5–15.2 (12.1 ± 1.4)	11.8–15.7 (13.3 ± 1.1)	13.0–16.8 (14.8 ± 0.9)
Macrogametocyte			
Infected erythrocyte			
Length	14.4–17.3 (15.6 ± 0.8)	12.9–16.9 (15.2 ± 0.7)	12.2–15.8 (13.8 ± 0.9)
Width	5.8–7.7 (6.9 ± 0.5)	6.0–7.5 (6.7 ± 0.5)	6.9–8.4 (7.8 ± 0.5)
Area	65.3–91.4 (77.8 ± 7.1)	64.7–94.6 (76.0 ± 6.8)	77.4–100.9 (86.2 ± 6.1)
Infected erythrocyte nucleus			
Length	5.9–7.5 (6.7 ± 0.5)	6.0–7.0 (6.3 ± 0.4)	3.7–5.8 (4.6 ± 0.5)
Width	2.1–2.7 (2.3 ± 0.2)	2.1–3.4 (3.0 ± 0.3)	2.9–3.9 (3.4 ± 0.3)
Area	8.4–13.1 (10.1 ± 1.0)	10.1–14.8 (10.0 ± 1.0)	11.1–15.1 (12.8 ± 1.2)
Gametocyte			
Length	18.8–31.5 (22.8 ± 3.2)	17.7– 28.7 (22.0 ± 2.2)	16.2–21.2 (18.8 ± 1.3)
Width	1.9–2.7 (2.4 ± 0.3)	2.2–2.8 (2.4 ± 0.2)	2.0–3.7 (2.8 ± 0.4)
Area	42.4–61.8 (48.4 ± 4.3)	39.6–69.7 (52.0 ± 4.8)	44.8– 63.6 (52.6 ± 5.4)
Gametocyte nucleus			
Length	2.0–3.7 (2.8 ± 0.4)	2.0–3.9 (2.9 ± 0.4)	–
Width	1.2–2.8 (2.0 ± 0.3)	1.9–3.6 (2.5 ± 0.4)	–
Area	3.1–5.3 (4.2 ± 0.6)	3.6–6.1 (4.9 ± 0.7)	–
Pigment granules	6.0–14.0 (9.4 ± 2.4)	7–18 (12 ± 2.7)	11.0–19.0 (14.5 ± 1.7)
NDR 2	0.7–1.1 (0.9 ± 0.1)	0.8–1.1 (0.9 ± 0.1)	0.4–0.9 (0.6 ± 0.2)
Microgametocyte			
Infected erythrocyte			
Length	13.1–16.7 (15.1 ± 0.8)	13.9–16.6 (15.0 ± 0.6)	12.2–14.4 (13.4 ± 0.5)
Width	6.0–7.8 (6.9 ± 0.4)	6.0–7.9 (6.8 ± 0.4)	6.5–8.8 (7.7 ± 0.5)
Area	62.7–95.1 (81.7 ± 8.8)	63.8–93.6 (75.5 ± 6.6)	65.8–93.6 (82.4 ± 6.5)
Infected erythrocyte nucleus			
Length	5.6–7.7 (6.6 ± 0.6)	6.0–7.7 (6.1 ± 0.4)	4.2–5.8 (4.9 ± 0.4)
Width	1.9–2.8 (2.3 ± 0.2)	2.0–3.6 (3.5 ± 0.2)	2.8–3.7 (3.3 ± 0.2)
Area	8.9–12.7 (10.5 ± 1.0)	10.0–13.8 (11.0 ± 1.2)	10.1–15.1 (12.5 ± 1.4)
Gametocyte			
Length	19.0–28.7 (23.9 ± 3.2)	18.8– 28.0 (21.0 ± 2.1)	17.9–24.0 (20.9 ± 1.5)
Width	1.9–3.4 (2.5 ± 0.3)	1.62–2.8 (2.4 ± 0.2)	1.6–3.6 (2.6 ± 0.5)
Area	42.3–61.5 (52.4 ± 5.4)	38.6–68.7 (50.0 ± 5.1)	38.2–66.6 (50.9 ± 6.7)
Gametocyte nucleus			
Length	5.8–8.1 (7.0 ± 0.7)	– 3	–
Width	1.8–2.6 (2.1 ± 0.2)	–	–
Area	9.6–21.9 (15.1 ± 3.1)	–	–
Pigment granules	7–19 (13.4 ± 3.1.)^4^	8–21 (14 ± 3.2)	13.0–25.0 (18.8 ± 3.1)
NDR	0.7–1.2 (0.8 ± 0.1)	0.8–1.1 (0.9 ± 0.1)	0.4–1.1 (0.7 ± 0.2)

1 All measurements (*n* = 21) are given in micrometres. Minimum and maximum values are provided, followed in parentheses by the arithmetic mean and standard deviation.

2 NDR = nucleus displacement ratio according to Bennett and Campbell (1972).

3 Microgametocyte nuclei were markedly diffuse and difficult to measure.

4 Pigment granules were difficult to calculate because they were masked by densely stained volutin.

The main diagnostic characters of *H. nisi* from *A. nisus* coincide with former descriptions [44, 53]. Molecular characterisation of this pathogen was developed for the first time, linking *H. nisi* to the *cytb* lineage hACCNIS06. The main features of the blood stages of this lineage are as follows.

Macrogametocytes and microgametocytes grow around the nucleus of infected erythrocytes, they markedly enclose the nucleus with their ends but do not displace or only slightly displace it laterally (Figs. 1a–1h). Young and growing gametocytes tend to not adhere to the erythrocytes’ nuclei (Figs. 1b, 1e). The central part of the advanced growing gametocytes is closely appressed to the erythrocyte’s envelope, but the ends of the gametocytes usually do not (Figs. 1b, 1c, 1g). Advanced growing gametocytes often do not adhere to the erythrocyte’s nucleus (Figs. 1b, 1e), but forms adhering to the nucleus are also common (Figs. 1c, 1f, 1g). Fully grown gametocytes are appressed to the nucleus and envelop of infected erythrocytes (Figs. 1d, 1h); they are circumnuclear and often completely encircle the nucleus (Figs. 1d, 1h) and can occasionally even occupy the entire cytoplasmic space in infected erythrocytes. The cytoplasm of the macrogametocytes is granular in appearance and contains a few small vacuoles (Figs. 1b, 1h). Volutin granules are abundant and clumped; they obscure pigment granules (Figs. 1b–1d, 1f–1h). The outline of the gametocytes varies markedly; it can be even, slightly wavy, or ameboid. The macrogametocytes’ nuclei are compact, variable in form, and median or sub-median in position (Figs. 1a, 1b). The pigment granules are predominantly oval, sometimes roundish, of medium size (0.5–1 μm), randomly scattered throughout the cytoplasm. The configuration of microgametocytes (Figs. 1e–1h) resembles that of the macrogametocytes with the usual sexual dimorphic characters, which are the paler stained cytoplasm, the diffuse centrally located nucleus and the predominant gathering of pigment granules close to the ends of the gametocytes (Figs. 1f–1h). Voucher blood preparation of *H. nisi* lineage hACCNIS06 (accession no. MNHN-IR-2023-01, 12 September 2013, HFS Goupil connexion, Hérault, France, collected by Dr. DVM Marie-Pierre Puech) was deposited in the Muséum National d’Histoire Naturelle, Paris. Representative DNA sequences: Mitochondrial *cytb* lineage hACCNIS06 (GenBank accession OR078933 and OR078931).

**Figure 2:**
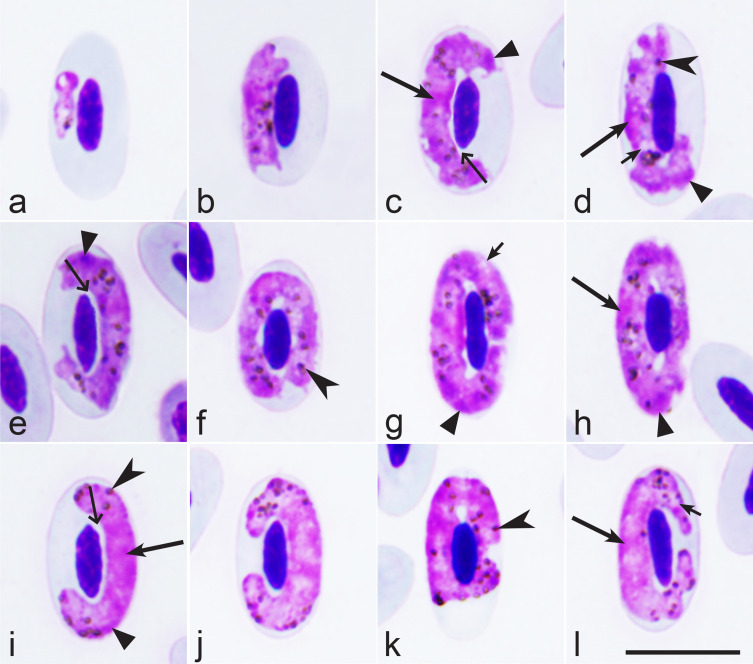
*Haemoproteus nisi* (lineage hCIAE08) from the blood of Western marsh harrier *Circus aeruginosus*: a – young gametocytes, b–h – macrogametocytes, i–l – microgametocytes. Long simple arrows – nuclei of parasites. Short simple arrows – vacuoles. Simple arrowhead – pigment granules. Triangle arrowheads – clamps of volutin. Simple wide long arrows – spaces between gametocytes and erythrocyte nuclei. Giemsa-stained thin blood films. Scale bar = 10 μm.

Morphological characterisation of *H. nisi* Peirce and Marquiss, 1983, the parasite lineage hCIAE08 found in an additional (non-type) host *C. aeruginosus* (lineage hCIAE08, Figs. 2a–2l, Table 1).

The main diagnostic characters of *H. nisi* from *C. aeruginosus* coincide with former descriptions [44, 53]. Molecular characterisation of the parasite lineage hCIAE08 was developed for the first time. The main features of hCIAE08 blood stages are as follows.

Macrogametocytes and microgametocytes grow around the nucleus of infected erythrocytes, they markedly enclose the nucleus with their ends but do not displace or only slightly displace it laterally (Figs. 2a–2l). The central part of the growing gametocytes predominantly adheres to the erythrocyte envelope but the ends of the gametocyte usually do not (Figs. 2c–2f, 2i–2k); the advanced growing gametocytes tend to adhere to nuclei of infected erythrocytes (Figs. 2b, 2d), but gametocytes not adhering to the nuclei were also seen (Figs. 2c, 2e, 2i). Fully grown gametocytes are circumnuclear; they often nearly completely or completely encircle the nuclei (Figs. 2f–2h) and can occasionally occupy the entire cytoplasmic space in infected erythrocytes. The cytoplasm of the macrogametocyte is heterogeneous in appearance and contains a few small vacuoles (Figs. 2d, 2g, 2l); volutin is present and seen as densely stained clamps of variable shape (Figs. 2d, 2e, 2g, 2h, 2i); the volutin might obscure the pigment granules, but not noticeably (Fig. 2h). The outline of gametocytes varies markedly; it can be even, slightly wavy or ameboid. The nuclei of the macrogametocyte are compact, variable in form, and median or sub-median in position (Figs. 2c, 2d, 2h). The pigment granules are roundish or oval, of medium size (0.5–1 μm), and randomly scattered throughout the cytoplasm (Figs. 2d, 2f, 2i, 2k). The configuration of the microgametocytes (Figs. 2i–2l) resembles that of the macrogametocytes with the usual sexual dimorphic characters, which are the paler stained cytoplasm, diffuse centrally located nuclei (Figs. 2i, 2l) and the predominant gathering of pigment granules close to ends of the gametocytes (Figs. 2i, 2j, 2l). Volutin is less evident in microgametocytes compared to macrogametocytes (compare Figs. 2e, 2g, 2h and 2i). Voucher blood preparation of *H. nisi* lineage hCIAE08 (accession no. MNHN-IR-2023-02, 4 April 2019, HFS Goupil connexion, Hérault, France, collected by Dr DVM Marie-Pierre Puech) was deposited in the Muséum National d’Histoire Naturelle, Paris. Representative DNA sequences: Mitochondrial *cytb* lineage hCIAE08 (GenBank accession OR078934).

*Haemoproteus* gametocytes observed in blood smears of the two *C. aeruginosus* infected with the parasite lineage hCIAE08 were morphologically similar to *H. nisi* hACCNIS06, but some differences were seen, mainly regarding the morphology of the volutin, which was more granular in appearance and more densely stained in parasites of the lineage hACCNIS06 (compare Fig. 1 and Fig. 2).

### Description of *Haemoproteus multivacuolatus* n. sp.

**Figure 3:**
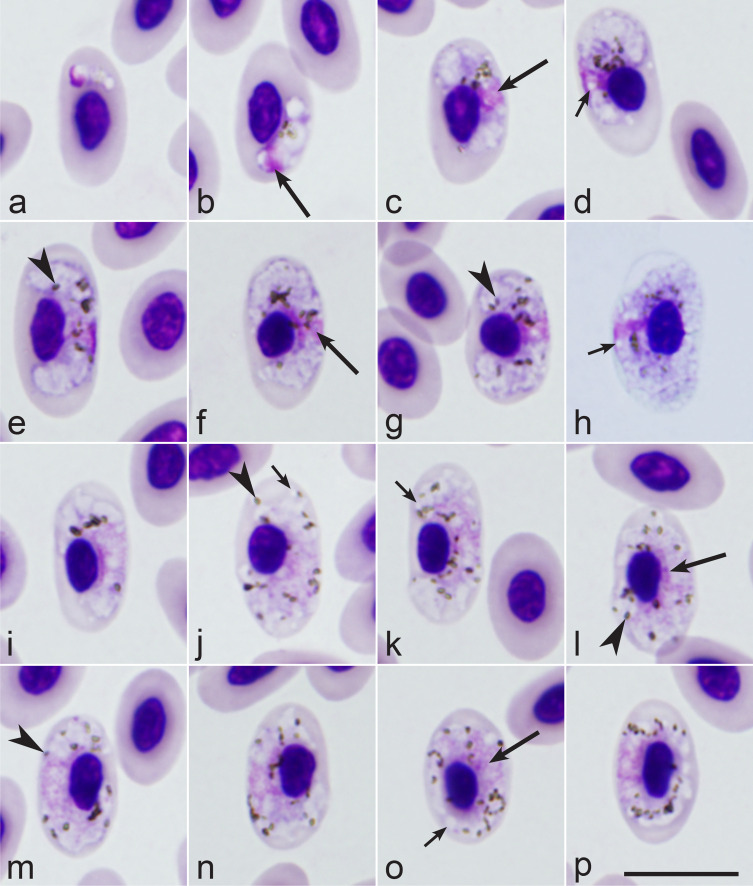
*Haemoproteus multivacuolatus* n. sp. (lineage hBUBT1) from the blood of the Common buzzard *Buteo buteo*: a–d – young gametocytes, e–h – macrogametocytes, i–p – microgametocytes. Long simple arrows – nuclei of parasites. Short simple arrows – vacuoles. Simple arrowhead – pigment granules. Triangle arrowheads – volutin granules. Note that due to marked vacuolisation, the cytoplasm of macrogametocytes stains relatively pale and looks similar to microgametocytes based on the intensity of staining. Giemsa-stained thin blood films. Scale bar = 10 μm. All images were from the hapantotype preparation.

*Haemoproteus multivacuolatus* n. sp. (lineage hBUBT1, Figs. 3a–3p, Table1).

urn:lsid:zoobank.org:act:22936DEA-BBFD-4FEA-9CB1-B64138A17035

Type-host: Common buzzard *Buteo buteo* (Accipitriformes).

Type locality: Osnabrück, Lower Saxony, Germany.

Type specimens: Hapantotype (intensity of parasitemia is 8.3%, *Buteo buteo,* approximately 4-week-old nestling, Osnabrück, Lower Saxony, Germany, N52.12535° E8.37812°, coll. N. Chakarov, July 2, 2019, *Leucocytozoon toddi* lMILANS04 is present) was deposited in the Muséum National d’Histoire Naturelle, Paris (accession no. MNHN-IR-2023-03). Parahapantotypes (no. 49491NS, a duplicate of the hapantotype and no. 49492NS, intensity of parasitemia 12.2%, *B. buteo,* Austria, *Leucocytozoon toddi* lMILANS04 is present) were deposited in the Nature Research Centre, Vilnius.

Additional material: Voucher blood films were deposited in the Muséum National d’Histoire Naturelle, Paris (accession no. MNHN-IR-2023-04).

Representative DNA sequences: Mitochondrial *cytb* lineage hBUBT1 (GenBank accession OR078932 and OR078930).

Distribution: Gametocytes were found in the type host *B. buteo* in Austria, Germany, and France. Transmission probably occurs throughout the breeding range of *B. buteo.* The lineage hBUBT1 was also found in five *A. nisus* individuals from France. However, the presence of gametocytes was not documented, so it remains unclear if the parasite completes the life cycle in this bird species.

Etymology: The species name reflects the markedly vacuolated appearance of the cytoplasm in both macrogametocytes and microgametocytes, resulting in minor differences between these types of gametocytes regarding the staining intensity (compare Figs. 3e–3h and Figs. 3i–3p).

Young gametocytes (Figs. 3a–3d) were seen anywhere in infected erythrocytes but more often occupied polar or subpolar positions in the host cells (Figs. 3a, 3b). As the gametocytes grow, they adhere to the nuclei of the erythrocytes and extend along the nuclei; they often assume asymmetrical positions regarding the nuclei, and this often leads to the appearance of comma-like gametocyte forms (Figs. 3c, 3d). Growing gametocytes often do not adhere to the erythrocyte envelope (Fig. 3c).

Macrogametocytes (Figs. 3e–3h; Table 1) are markedly vacuolated, lack volutin granules, and the cytoplasm stains relatively pale in comparison to microgametocytes (compare Figs. 3e–3h and Figs. 3i–3p), which is a characteristic feature of this parasite. Gametocytes extend along nuclei of erythrocytes (Fig. 3e); they markedly enclose the erythrocyte nuclei with their ends (Figs. 3f, 3g) and finally completely encircle the nuclei (Fig. 3h). Advanced and fully grown gametocytes are closely appressed to the nucleus and envelope of the erythrocytes; however, they usually do not fill poles of erythrocytes completely, resulting in the presence of more or less visible unfilled spaces on the poles (Figs. 3f–3h). The outline of gametocytes is predominantly wavy, sometimes slightly ameboid or even. Parasite nucleus median or submedian in position, markedly variable in form and outline; its boundaries were poorly visible (Figs. 3e–3h), which is a rare character in macrogametocytes of avian haemoproteids. Pigment granules are predominantly oval, sometimes roundish, of small (<0.5 μm) and average (0.5–1 μm) size, randomly scattered throughout the cytoplasm. Nuclei of infected erythrocytes only slightly displaced laterally (Figs. 3e–3h); however, advanced growing gametocytes (Figs. 3e–3g) and fully grown gametocytes (Fig. 3h) cause marked enlargement of infected erythrocytes in length (*p* < 0.01) and particularly in area (*p* < 0.001) in comparison to uninfected erythrocytes (Table 1), a characteristic feature of this species, which was readily recognisable in blood films even without statistical analysis (see Figs. 3e, 3g, 3k, 3m for comparison of the size of the infected and uninfected erythrocytes).

Microgametocytes (Figs. 3i–3p; Table 1) are similar to macrogametocytes in general configuration and other features. Gametocyte nuclei are markedly diffuse, of irregular form. The outline of nuclei is poorly recognisable, so the nuclei were difficult to measure.

Taxonomic remarks: Four distinct morphospecies of *Haemoproteus* parasites were described in birds of the order Accipitriformes. These are *H. elani* Mello, 1935, *H. buteonis* Wingstrand, 1947, *H. janovyi* Greiner, Mundy, 1979, and *H. nisi* Peirce, Marquiss, 1983. *Haemoproteus multivacuolatus* n. sp. can be readily distinguished from all these parasites, primarily due to remarkable hypertrophy of infected erythrocytes in area (Table 1; compare the appearance of infected and uninfected erythrocytes in Fig. 3). Slight enlargement of infected erythrocytes by fully grown gametocytes might occur in other haemoproteids, but this feature is not as distinct in other species parasitising accipitriform birds (Table 1). It is important to note that even growing gametocytes of *H. multivacuolatus* n. sp. induce marked enlargement of infected erythrocytes (Figs. 3d, 3e); that is not the case in any other haemoproteids parasitising accipitriform birds. Additionally, fully grown gametocytes of *H. buteonis* and *H. elani* are markedly halteridial [58], which is not the case in *H. multivacuolatus* n. sp.

It is important to note that the identification of *H. multivacuolatus* n. sp. might be difficult if only growing gametocytes are present in a sample. Such blood samples from *B. buteo* were common in this study. If parasitaemia is represented only by growing gametocytes (Figs. 3e, 3i–3k) but fully grown (circumnuclear) gametocytes (Figs. 3h, 3o, 3p) are absent, *H. multivacuolatus* n. sp. identification might be difficult due to similarities with fully grown gametocytes of *H. buteonis* and *H. elani*. Molecular characterisation would be particularly helpful in this case.

Circumnuclear fully grown gametocytes predominate in both lineages of *H. nisi* (hACCNIS06 and hCIAE08) (Figs. 1, 2) and *H. janovyi* [56]. *Haemoproteus multivacuolatus* n. sp. can be readily distinguished from both *H. nisi* lineages due to the lack of volutin and the marked vacuolisation of gametocyte cytoplasm in the former. Fully grown gametocytes of *H. janovyi* predominantly occupy all available space in infected erythrocytes and are also markedly pleomorphic in morphology [58]; both these characters are not features of *H. multivacuolatus* n. sp.

### Molecular analyses

A minimum of 300 reads that aligned to the reference sequence were obtained for amplicons 1 and 2, covering the whole 478 bp *cytb* barcode sequence, for all DNA barcodes of *Haemoproteus* parasites of Accipitriformes from France. The sequence analysis revealed five different *cytb* lineages; three of which belonged to *Haemoproteus* parasites (hBUBT1, hCIAE08, and hACCNIS06) and two to *Plasmodium* parasites (pTURDUS1, pBT7).

*Haemoproteus multivacuolatus* hBUBT1 was found in 53/53 (100%) *B. buteo* and 5/22 (22.7%) *A. nisus, H. nisi* hACCNIS06 was found in 22/22 (100%) *A. nisus*, and *H. nisi* hCIAE08 was found in 2/2 (100%) *C. aeruginosus* and 1/53 (1.9%) *B. buteo*. Some individuals had co-infections with *Plasmodium circumflexum* pTURDUS1, which was found in 7/22 (31.8%) *A. nisus* and 1/53 (1.9%) *B. buteo*, and *P. circumflexum* pBT7, which was found in 2/22 (9.1%) *A. nisus* and 5/53 (9.4%) *B. buteo*. Mixed infections with different *Haemoproteus* parasite lineages were present in 1/53 (1.9%) *B. buteo* (hBUBT1/hCIAE08 lineages), 5/22 (22.7%) *A. nisus* (hBUBT1/hACCNIS06 lineages), and none of the 2 *C. aeruginosus* (Table 2).

**Table 2:** *H. multivacuolatus* n. sp. hBUTBUT01, *H. nisi* hACCNIS06 and *H. nisi* hCIAE08, their accipitriform host species and their overall prevalence in France, Austria and Germany.

*Haemoproteus* morphospecies	Host species	Overall prevalence
*H. multivacuolatus* n. sp. hBUBT1	Common Buzzard, *Buteo buteo* (France)	53/277 (19.1%)
	Eurasian Sparrowhawk, *Accipiter nisus* (France)	5/131 (3.8%)
	Common Buzzard, *Buteo buteo* (Austria)	26/70 (37.1%)
	Common Buzzard, *Buteo buteo* (Germany)	69/832 (8.3%)
*H. nisi* hACCNIS06	Eurasian Sparrowhawk, *Accipiter nisus* (France)	22/131 (16.8%)
	Eurasian Sparrowhawk, *Accipiter nisus* (Austria)	4/22 (18.2%)
*H. nisi* hCIAE08	Western Marsh Harrier, *Circus aeruginosus* (France)	2/4 (40%)
	Common Buzzard, *Buteo buteo* (France)	1/277 (4.4%)
	Western Marsh Harrier, *Circus aeruginosus* (Austria)	7/22 (31.8%)

The molecular screening of samples from Austria specifically targeted *H. nisi* species group. Sequences retrieved with other primer sets from the same sample were published previously [18]. For the present study, we screened 171 Accipitridae from 15 species and found 42 (24.6%) individuals infected with *Haemoproteus* parasites of the Accipitridae group. *Haemoproteus multivacuolatus* hBUBT1 was detected in 26/70 *B. buteo* (37.1%), *H. nisi* hACCNIS06 was found in 4/22 *A. nisus* (18.2%), and *H. nisi* hCIAE08 in 7/22 *C. aeruginosus* (31.8%). Three more *cytb* lineages were found in a few individuals only: *Haemoproteus* sp. hACCNIS07 was found in one *A. gentilis* (11.1%), one *A. nisus* (4.6%), and one *C. aeruginosus* (4.6%). One *A. gentilis* (11.1%) and one *A. nisus* (4.6%) featured co-infections with *Haemoproteus* sp. hACCNIS08 and *Haemoproteus* sp. hACCNIS09 (Table 2).

In Germany, 832 *B. buteo* nestlings were screened, of which 69 (8.3%) were infected with *H. multivacuolatus* n. sp. hBUBT1 (Table 2).

Of the six *Haemoproteus* lineages found in the present study, complete *cytb* barcode sequences were only published for the lineages hBUBT1 and hCIAE08. Lineage hBUBT1 was reported from single individuals of *B. buteo* in Germany [29] and Austria [18], who referred to hBUBT1 as “*Haemoproteus elani*”. Lineage hCIAE08 was published from one *C. aeruginosus* in Austria under the name “*Haemoproteus* aff. [affinis] *elani*” [18]. However, the morphology of the two parasite lineages had not been assessed previously. In the present study, hBUBT1 was linked to the new species *H. multivacuolatus* n. sp., and hCIAE08 and hACCNIS06 were linked to *H. nisi*. A partial sequence of lineage hACCNIS06 (ON375836, 424 bp) from *A. nisus* in Czechia was published by Svobodová *et al.* [51]. The latter authors also published a partial sequence of hACCNIS07 (ON375837, 384 bp) from the same host, which we found in one *A. gentilis*, *A. nisus*, and *C. aeruginosus* in Austria. Apart from the latter four lineages, we detected for the first time hACCNIS08 and hACCNIS09 in one *A. gentilis* and one *A. nisus*. For the lineages hACCNIS07, hACCNIS08, and hACCNIS09, blood smears were either not available or of insufficient quality.

### DNA haplotype network

**Figure 4:**
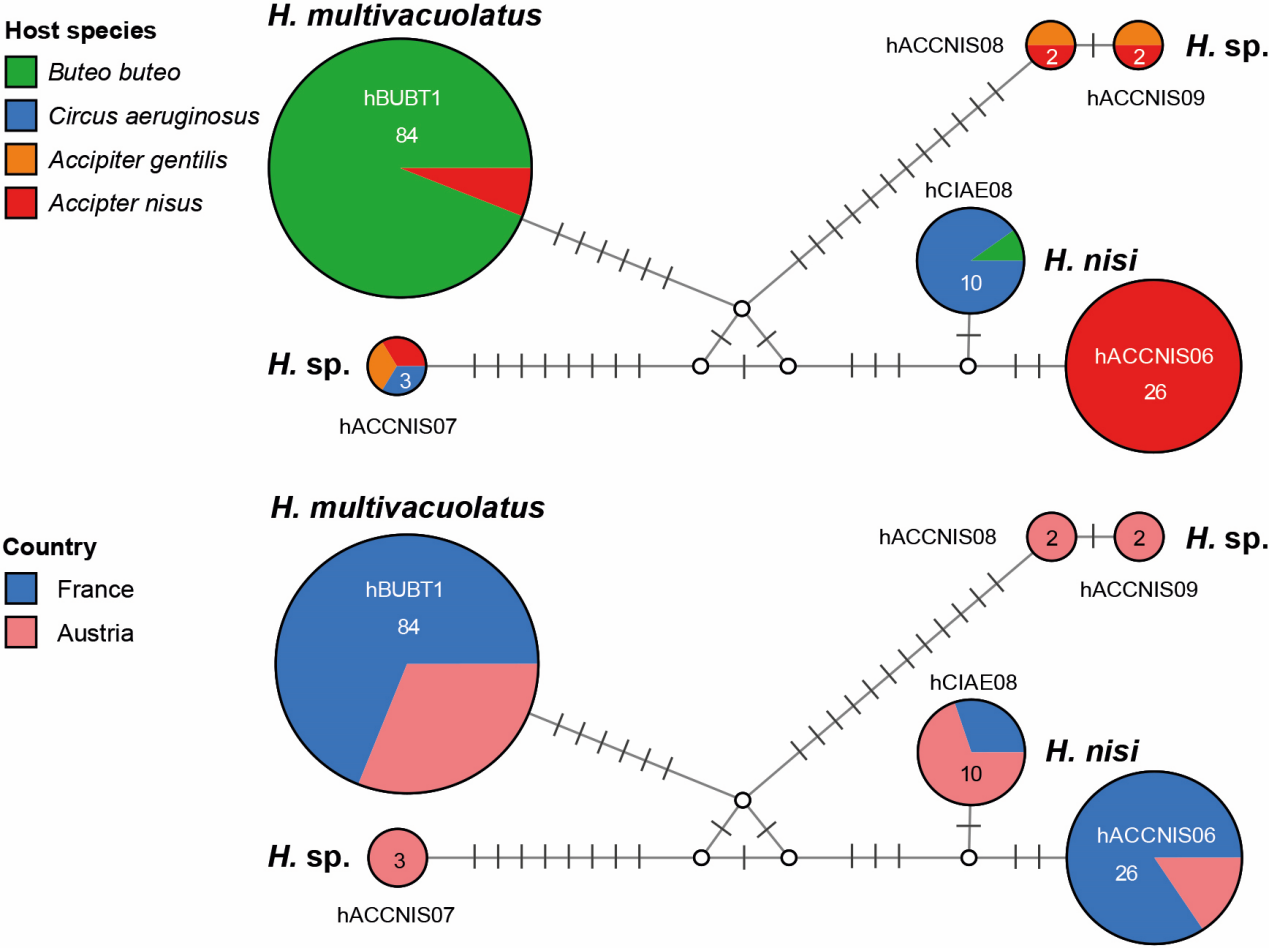
Median-Joining DNA haplotype network showing the host and geographic distribution of six *Haemoproteus nisi* group lineages (478 bp *cytb* sequences) found in accipitriform raptors from Austria and France.

A DNA haplotype network based on the 478 bp *cytb* barcode sequences identified in the birds from Austria and France, is presented in Figure 4. The six *Haemoproteus* lineages clustered into four subclades, differing by up to 22 bp from each other. The genetic distance between the four groups ranged from 2.4% to 4.5%. The first group includes *H. multivacuolatus* n. sp. hBUBT1, which was detected in 84 individuals (79 *B. buteo* and 5 *A. nisus*). The second group includes only *Haemoproteus* sp. hACCNIS07, which was found in single specimens of *A. gentilis*, *A. nisus*, and *C. aeruginosus* in Austria. The third group comprises *Haemoproteus* sp. hACCNIS08 and *Haemoproteus* sp. hACCNIS09, which were both found in single specimens of *A. gentilis* and *A. nisus* in Austria; the two lineages differ by 1 bp (0.2%) in the 478 bp *cytb* sequence. The last group includes *H. nisi* hACCNIS06, which was found in 26 *Accipiter nisus* from France and Austria, and *H. nisi* hCIAE08, which was found in 9 *C. aeruginosus* 1 *B. buteo* in Austria and France; the two *cytb* lineages differ by 3 bp (0.6%) from each other. The average genetic distance between the 478 bp *cytb* barcode sequence of lineages belonging to the *H. nisi* species group and other haemosporidian parasites (including other *Haemoproteus* species) was higher than 15%.

### Complete mitochondrial *cytb* sequences

Complete *cytb* gene sequences were obtained for three of the *Haemoproteus* lineages: *H. multivacuolatus* n. sp. hBUBT1, *H. nisi* hACCNIS06, and *H. nisi* hCIAE08. A total average of about 47,250 reads (minimum of 40,765 to a maximum of 54,480 reads) and an average coverage depth of about 7,400 reads (minimum of 6,630 to maximum of 8,110 reads) reads were obtained. The average molecular divergence between the complete *cytb* sequences of the *H. nisi* group and those of other haemosporidian parasites, including *Haemoproteus* spp., was congruent with the divergence of the *cytb* barcode sequence, with more than 15%. The *cytb* gene sequences (1100 bp) obtained from the French samples were deposited in GenBank (accession numbers OR078932, OR078933, and OR078934) and the MalAvi database. The *cytb* sequences (821 bp) obtained from the samples from Austria were also deposited in GenBank (accession numbers OR293360–OR293403) and the MalAvi database.

### Complete mitochondrial genomes

Mitochondrial genomes of 5959 bp were obtained for both *H. multivacuolatus* n. sp. hBUBT1 and *H. nisi* hACCNIS06. The mitochondrial genomes of the two parasite species diverged by 2.8%. They showed the same genome organisation commonly shared with mitochondrial genomes of other haemosporidian parasites, characterised by a linear element of ∼6 kbp containing the three protein-coding genes, *cytb*, *cox1*, and *cox3*, and two highly fragmented (small and large subunits) ribosomal RNA (rRNA) genes [21]. The two mitochondrial genome sequences were deposited in GenBank under accession numbers OR078930 and OR078931.

### Phylogenetic analyses

**Figure 5:**
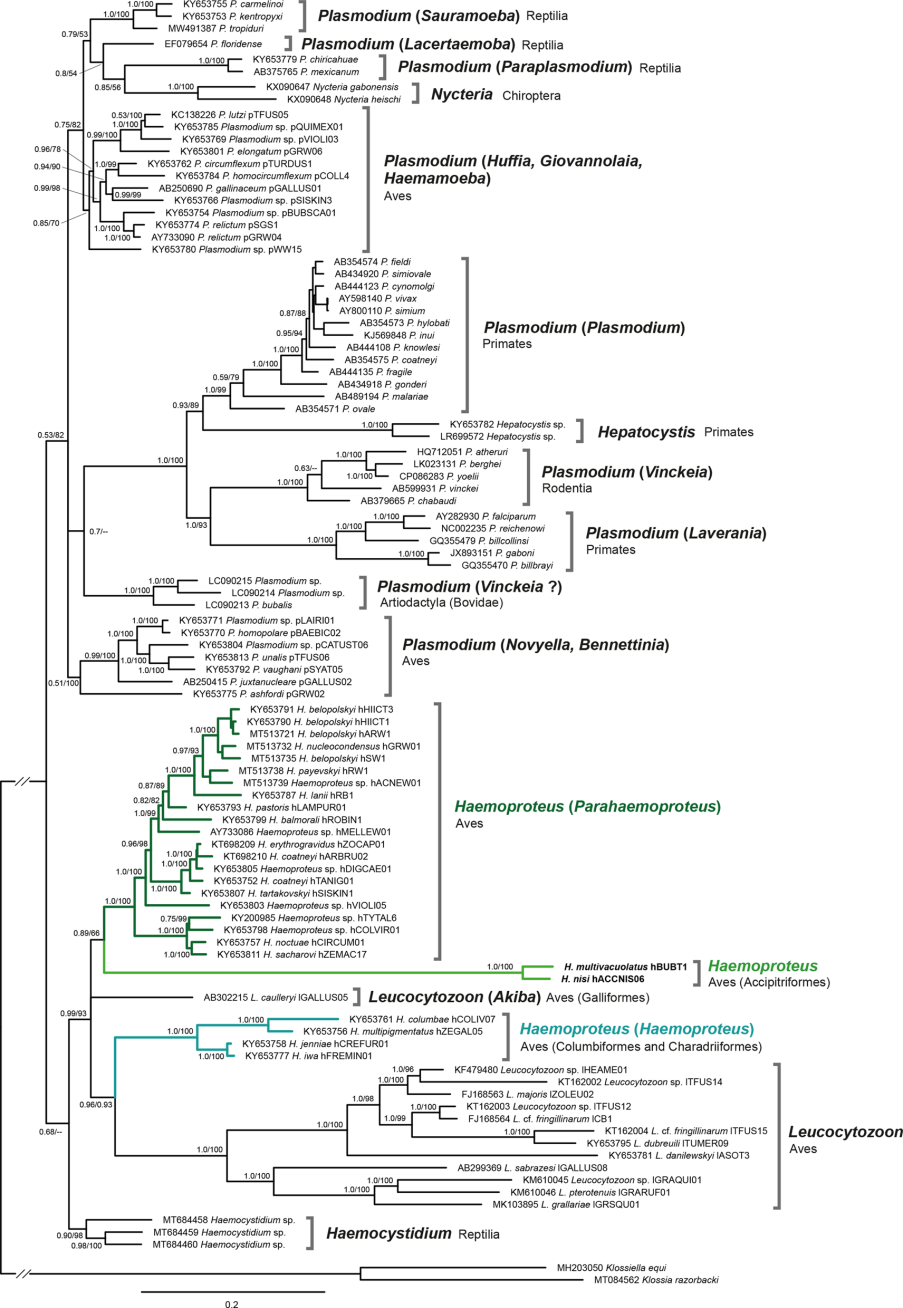
Bayesian Inference tree calculated with complete *cox1* (1428 bp), *cox3* (753 bp), and *cytb* (1127 bp) sequences of haemosporidian parasites and *Klossiella equi* (MH203050) and *Klossia razorbacki* (MT084562) as the outgroup. Bayesian posterior probabilities and Maximum Likelihood bootstrap values are indicated at most nodes. The scale bar indicates the expected number of substitutions per site according to the model of sequence evolution applied.

**Figure S1:**
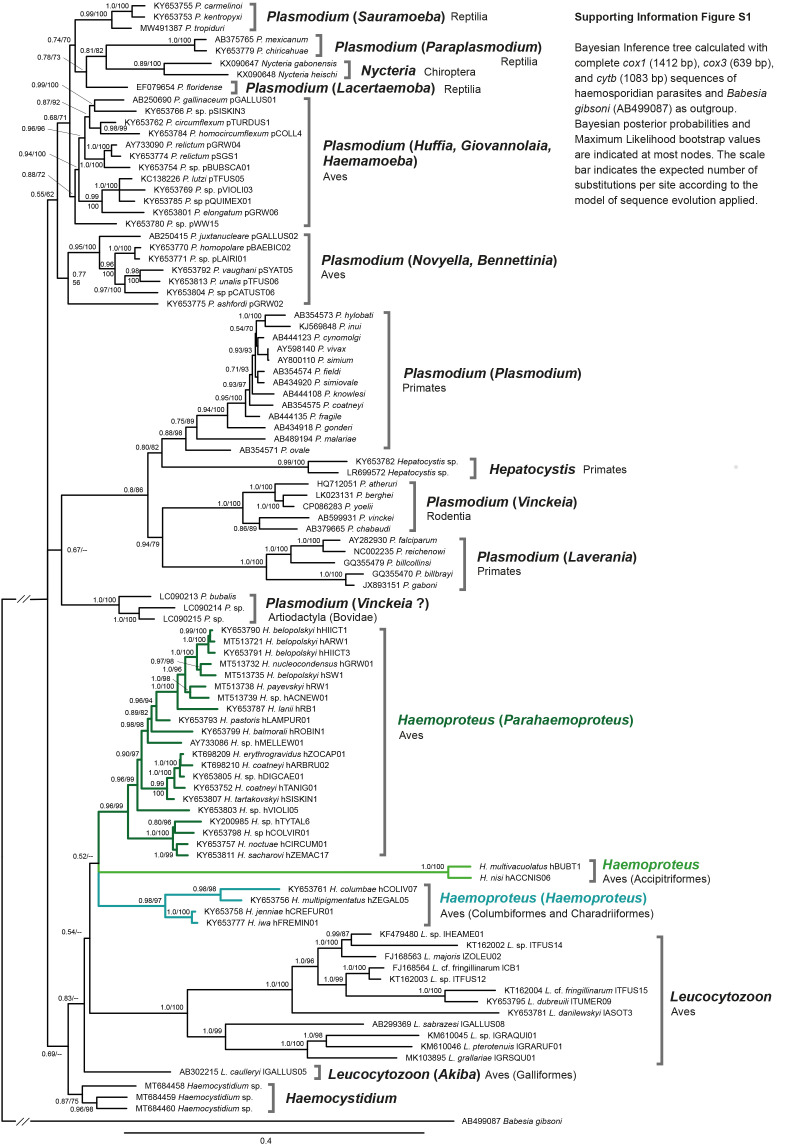
Bayesian Inference tree calculated with complete *cox1* (1412 bp), *cox3* (639 bp), and *cytb* (1083 bp) sequences of haemosporidian parasites and *Babesia gibsoni* (AB499087) as the outgroup. Bayesian posterior probabilities and Maximum Likelihood bootstrap values are indicated at most nodes. The scale bar indicates the expected number of substitutions per site according to the model of sequence evolution applied.

The phylogenetic trees were rooted with sequences of *Klossiella equi* (MH203050) and *Klossia razorbacki* (MT084562), whose mitochondrial genomes are most similar to those of haemosporidian parasites compared to those of apicomplexan parasites of other orders (Fig. 5). *Haemoproteus multivacuolatus* hBUBT1 and *H. nisi* hACCNIS06 formed a clade on a long branch with maximum support (BI posterior probability: 1.0/ML bootstrap support: 100), which was part of a highly supported clade (0.99/93) including *Haemoproteus* (*Haemoproteus*), *Haemoproteus* (*Parahaemoproteus*), *Leucocytozoon* (*Akiba*) *caulleryi*, and *Leucocytozoon*. Within this clade, the *H. nisi*/*H. multivacuolatus* group clustered with *Haemoproteus* (*Parahaemoproteus*) in a subclade with moderate support (0.89/66), *Haemoproteus* (*Haemoproteus*) clustered with *Leucocytozoon* with high support (0.96/0.93), and *L.* (*Akiba*) *caulleryi* formed a third subclade. In the additional phylogenetic trees reconstructed with *Babesia gibsoni* (AB499087) as outgroup (Fig. S1), *Haemoproteus multivacuolatus* hBUBT1 and *H. nisi* hACCNIS06 also formed a clade on a long branch with strong support (BI posterior probability: 1.0/ML bootstrap support: 100). This clade clustered with *Haemoproteus* and *Parahaemoproteus* parasite clade with low support (0.52/–).

## Discussion

Despite several records of *Haemoproteus* infections in accipitriform raptors [18, 24, 29, 42, 51], these parasite lineages have not been characterised morphologically so far. For the present study, we screened 1080 accipitriform raptors from France, Austria, and Germany for *Haemoproteus* parasites. The sample from France included 77 Accipitridae of the species *B. buteo*, *A. nisus*, and *C. aeruginosus*, which were all previously confirmed positive for *Haemoproteus* infections based on blood smear screenings on a total of 413 Accipitridae birds (18.6%). The sample from Austria comprised 171 birds from 15 Accipitridae species, of which 42 individuals (24.6%) were infected with *Haemoproteus* parasites. The sample from Germany included 832 *B. buteo* nestlings, of which 69 (8.3%) were *Haemoproteus*-positive. Different methods were used to screen the samples from the three countries because they were originally part of three independent studies. However, the authors decided to combine the results because they covered the same subject.

The central aim of the present study was to gather new sequence data of these *Haemoproteus* parasites and to characterise the morphology of their blood stages. *Haemoproteus nisi*, which was previously reported mainly from *Accipiter* spp. and *Circus* spp. [53], was characterised molecularly and linked to the *cytb* lineages hACCNIS06 and hCIAE08. *Haemoproteus multivacuolatus* n. sp. was described and linked to the *cytb* lineage hBUBT1, which is common in *B. buteo*.

The development of new primers was essential because those used in the standard PCR protocol by Hellgren *et al.* [19] and most other published primer sets do not match the *cytb* of parasites belonging to the *H. nisi* species group. Since the original studies were conducted independently, three different protocols were used to sequence the *cytb* barcode region of parasites belonging to the *H. nisi* group.

It is important to note that co-infections with haemosporidian parasites of one genus or several genera are common in wildlife, which often results in an electropherogram containing double peaks that are hard to untangle when PCR products are sequenced directly using Sanger sequencing. Deep sequencing with Illumina or other NGS methods can help avoid this problem. Another option for avoiding the amplification of multiple lineages in parallel is using primers that specifically target certain groups of parasites. Both methods were successfully applied in the present study.

The PCR and sequencing protocol for the samples from France also detected co-infections with two *Plasmodium circumflexum* lineages, pTURDUS1 and pBT7, but blood stages of the latter were not detected by blood-film microscopy, probably due to low parasitaemia. *Plasmodium circumflexum* is a generalist pathogen that is widespread globally and was found particularly often in Paridae, but also in birds of other families and orders, including Accipitridae [59]. Although few molecular records (less than 30) of *P. circumflexum* pTURDUS and pBT7 were reported from Accipitridae hosts, these were two of the most frequently detected *Plasmodium* lineages in accipitriform raptors [18].

However, the focus of the present study was on parasites belonging to the *H. nisi* group, which were only detected in accipitriform raptors so far. In total, we identified six *Haemoproteus* lineages, three of which were common and almost host-specific. The lineage *H. multivacuolatus* n. sp. hBUBT1 was mainly found in *B. buteo*, *H. nisi* hACCNIS06 in *Accipiter nisus*, and *H. nisi* hCIAE08 in *C. aeruginosus*. The other three lineages were found only in a few birds, *Haemoproteus* sp. hACCNIS07 in one *A. nisus*, one *A. gentilis*, and one *C. aeruginosus* in Austria (present study) and in *A. nisus* in Czechia [51] and *Haemoproteus* sp. hACCNIS08 and hACCNIS09 in one *A. gentilis* and one *A. nisus* in Austria (present study). Previously, sequences of *H. nisi* group parasites have been reported from Europe and Northern America [18, 24, 29, 43, 51], but in most cases the published sequences did not cover the entire 478 bp *cytb* barcode region. Ishak *et al.* [24] identified several different *Haemoproteus* lineages in accipitriform raptors from North America, which covered 242 bp of the *cytb* barcode region. Sequences identical to hBUBT1 in the 242 bp section were found in *Buteo jamaicensis* (FJ966919, FJ966924, FJ966925, FJ966926) and *Accipiter cooperi* (FJ966925) and were referred to as *H. nisi*. One *A. cooperi* (FJ966921) was infected with a parasite lineage identical to hCIAE08, and some *A. cooperi* (FJ966920, FJ966923) were infected with another lineage that would form a fifth subclade in the *H. nisi* group. It should be noted that the sequences matching hBUBT1 in the 242 bp MalAvi region belonged to four lineages differing by a single bp from each other in the remaining 270 bp. Outlaw and Ricklefs [43] also screened *Buteo jamaicensis* from the USA and sequenced a 291 bp section of *cytb*, which only covers 25 bp of the DNA barcode region. Most of the sequences (GQ141607, GQ141611, GQ141613, GQ141615–GQ141617, and GQ141628) are identical to the complete *cytb* sequence of hBUBT1 generated in the present study. Two other sequences (GQ141628 and GQ141629) differ by 1 bp from hBUBT1, but are identical to a lineage (FJ966919) found in the same host species by Ishak *et al.* [24]. Outlaw and Ricklefs [43] did not assign these lineages to any of the known genera because of their uncertain phylogenetic position. Ishak *et al.* [24] referred to hBUBT1 and related lineages as *H. elani* due to the similarity of gametocytes, which is probably not correct because this parasite species was originally described from *Elanus caeruleus* in western India by de Mello [34] and is likely a distinct parasite species. Krone *et al.* [29] and Harl *et al.* [18] found hBUBT1 in *B. buteo* from Germany and Austria, respectively, but morphological analyses were not conducted.

*Haemoproteus multivacuolatus* n. sp. hBUBT1 can be readily distinguished from other haemoproteids parasitising accipitriform birds, particularly because the fully grown gametocytes are circumnuclear, which markedly enlarges the infected erythrocytes (Figs. 3h, 3l). The proportion of young and growing gametocytes depends on the stage of parasitaemia, and the fully grown gametocytes are not always available in wildlife samples. This is an obstacle for distinguishing *H. multivacuolatus* n. sp. hBUBT1 from *H. elani* and *H. buteonis*. Molecular data (Fig. 4) show that *H. multivacuolatus* n. sp. predominates in *B. buteo*. *Haemoproteus buteonis* is rare in this bird species. The latter parasite also infects *A. nisus* [53, 58]. *Haemoproteus buteonis* was synonymised with *H. elani* by Peirce *et al.* [44], but Valkiūnas [53] suggested considering *H. buteonis* a distinct species until the variability of gametocytes is studied further. Molecular characterisation of both species is still absent. Redescription and molecular characterisation of *H. elani* is needed, preferably from its vertebrate type-host the black-winged kite *E. caeruleus* sampled close to the type locality in Goa, India.

In the present study, the *cytb* lineages hACCNIS06 and hCIAE08 were linked to the morphospecies *H. nisi*. The lineage hACCNIS06 was first detected in Czechia (ON375836) in *A. nisus* [51], but a combined molecular and morphological characterisation has not been done so far. The sequences of the latter study were not included in the DNA haplotype networks (Fig. 4) because they did not cover the entire *cytb* barcode region. In France and Austria, hACCNIS06 was also exclusively recorded in *A. nisus*. According to Valkiūnas [53], *H. nisi* has been found in a variety of *Accipiter* spp., *Circus* spp., and a few species of other genera. Ishak *et al.* [24] found a parasite lineage in *A. cooperii* in the United States that differed by 2 bp (0.4%) and 3 bp (0.6%) from hACCNIS06 and hCIAE08 in the 512 bp *cytb* fragment published (FJ966921, covering only 242 bp of the DNA barcode sequence.

Lineage hCIAE08 was recently reported from *C. aeruginosus* in Austria [18], and referred to as *H.* aff. *elani* but the morphology of the blood stages could not be studied. In this study, gametocytes of the lineage hCIAE08 were observed in two *C. aeruginosus* individuals and were attributed to the *H. nisi* group as a morphological variant. This parasite, which morphologically resembles *H. nisi* and is genetically similar, is referred to as a variant belonging to the *H. nisi* group at this stage of research. The two lineages only differ by 3 bp (0.6%) in the *cytb* barcode sequence, but were found in different host species, suggesting that they might belong to two separate parasite species. To clarify the taxonomic status of the parasite lineage hCIAE08, more information on the biology (blood and tissue stages, vectors) and nuclear markers are needed. The lack of data about vectors, sporogony, and exo-erythrocytic development of *Haemoproteus* parasites infecting accipitriform birds is problematic and this information would be crucial to better understand the true pathogenicity of these parasites [10].

The results of the present and previous molecular studies indicate that the diversity of *Haemoproteus* species in accipitriform raptors is larger than previously described using the traditional taxonomy characters based on morphology of blood stages. The majority of *cytb* lineages are not yet linked to morphotypes and might also represent distinct parasite species. This study shows that *H. nisi* is probably a group of closely related species or subspecies, which currently consist of the two lineages hACCNIS06 and hCIAE08.

The development of new diagnostic tools is essential to detect highly molecularly divergent parasites that might be undetectable by commonly used conventional tools [17]. Thus, it is likely that there are other parasites that remain undetected. In this case, the use of microscopy remains a useful diagnostic tool which allows the detection of a diversity of parasites not detectable with the available molecular tools and thus allows the design of appropriate diagnostic approaches to target these new pathogens.

The available molecular data show the presence of at least five distinct groups (or subclades) of *Haemoproteus* parasites in accipitriform raptors. The first group comprises *H. multivacuolatus* n. sp. hBUBT1 and a few similar *cytb* lineages (FJ966919, FJ966926, FJ966924) mostly found in *Buteo* spp. The second group includes the two *H. nisi* lineages hACCNIS06 and hCIAE08 (and FJ966921), which were mostly found in *Accipiter* spp. and *Circus* spp. The third group includes hACCNIS07 from *Accipiter* spp. and *Circus* spp., the fourth comprises hACCNIS08 and hACCNIS09 from *Accipiter* spp., and the fifth includes two lineages (FJ966920 and FJ966923) from *A. cooperi*. The morphology of parasites belonging to the latter three groups has not yet been assessed.

*Haemoproteus* parasites from accipitriform raptors form a unique phylogenetic clade, which seems to be specific to this bird order. These *cytb* sequences diverge by more than 15% from all other known *Haemoproteus*, *Plasmodium*, and *Leucocytozoon* lineages. The molecular divergence has led to the erroneous suggestion about possible attribution of these *cytb* lineages to *Plasmodium* parasites [29] or non-assignment to any of the three common avian genera [18, 43, 51]. A divergence of 15% in *cytb* is observed elsewhere between parasites of different genera. Thus, parasites of the *H. nisi* group might be classified as a separate subgenus or even genus when more information on their biology becomes available. Until more data are available on the formation of exo-erythrocytic stages in the bird hosts and on development and transmission in dipteran vectors, the current taxonomic status of these parasites is retained. Our study calls for this type of basic parasitology research, which might add new data on haemosporidian parasite biology and generate taxonomic characters. This could be used to develop classification within the order Haemosporida. Studies screening a broader range of potential host species and more individuals are needed to better understand the diversity, intraspecific genetic variation, host-parasite relationships, and geographical distribution of these *Haemoproteus* parasites.

## Supplementary Files

The Supplementary materials of this article are available at https://www.parasite-journal.org/10.1051/parasite/2023066/olm.
